# Upward and Northwest Range Shifts for Four Endemic Lamiaceae Medicinal Herbs in the Third Pole

**DOI:** 10.1002/ece3.71116

**Published:** 2025-03-18

**Authors:** Shou‐Kui Wang, Zhi‐Peng Li, Rui Wu, Hai‐Ling Qi, Hong Ke, Xiong‐Hui Huang, Ji‐Hua Zhou, Yong Tang, Jiang‐Hua Ran, Yong‐Qian Gao

**Affiliations:** ^1^ China Southern Power Grid EHV Transmission Company Guangzhou China; ^2^ CAS Key Laboratory for Plant Diversity and Biogeography of East Asia, Kunming Institute of Botany Chinese Academy of Sciences Kunming China; ^3^ University of Chinese Academy of Sciences Beijing China; ^4^ Yunnan Electric Power Design Institute Co., Ltd China Energy Engineering Group Kunming China; ^5^ Yunnan Forestry Technological College Kunming China

**Keywords:** alpine species, climate change, Lamiaceae, range shift, the third pole

## Abstract

In response to climate warming, alpine plants are migrating to higher elevations and latitudes to track suitable habitats. In mountainous systems, the contraction of land area toward mountaintops is causing plant habitats to shrink as plants migrate upwards. The Tibetan Plateau (TP) and its adjacent Himalaya–Hengduan Mountains (HHMs) constitute the world's highest flora, known as the “Third Pole” To predict the responses to climate change of alpine plants in the Third Pole, we utilized four endemic Lamiaceae alpine herbs as an Ecological Niche Modeling (ENM) based on comprehensive data comprising 740 occurrence records and 26 environmental variables using *Biomod2*. The primary results revealed that climate‐related factors, particularly temperature variability, shape the distribution patterns of the study species and drive them to migrate upward and northward in the future. The heterogeneous topography of the HHM and the TP leads to distinct distribution dynamics. The TP can provide substantial new potential distribution areas to mitigate habitat loss in the adjacent HHM under climate warming. Meanwhile, stable areas in the high‐elevation regions within HHM can serve as refugia to ensure species survival under climate change.

## Graphical abstract

To predict the responses to climate change of alpine plants in the Third Pole, the region with the highest flora, we used four endemic Lamiaceae alpine herbs as a model, carrying out the Ecological Niche Modeling analyses. The main results showed that, in the future, Lamiaceae species are projected to migrate upward and northward, and the Tibet Plateau is anticipated to become a migration destination for four Lamiaceae species, and the stable and suitable areas at the summits of the Himalaya–Hengduan Mountains region are crucial for ensuring the survival of alpine species amidst climate change. Our findings will not only help to improve our understanding of the impacts of climate change on alpine plants in the Third Pole, but will also provide a case study of Ecological Niche Modeling using a similar approach.

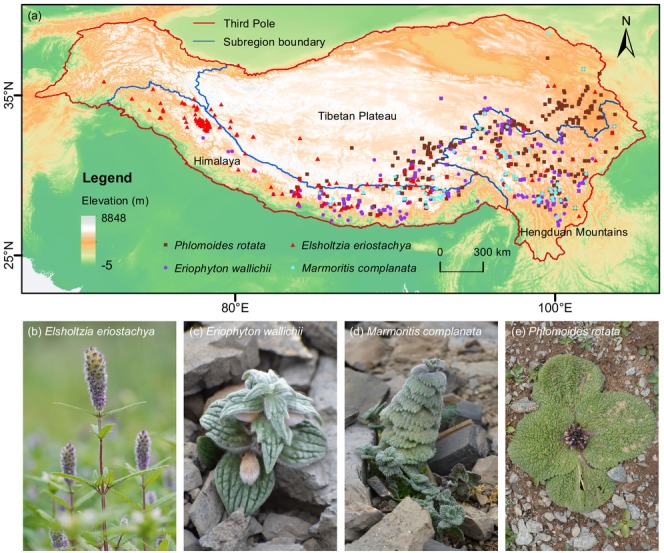

## 1 Introduction

Over the past century, global temperature has experienced a sharp increase and is projected to accelerate in the future (IPCC 2023). Rising temperature has exerted profound impacts on global ecosystems, putting numerous species and biodiversity hotspots under severe threat in recent decades (Urban 2015). Mountain systems, which harbor most of the world's biodiversity hotspots, have been identified as susceptible regions. Alpine areas are more vulnerable to climate change, and alpine species are considered to be at elevated risk (Engler et al. 2011; Nogués‐Bravo et al. 2007; Noroozi et al. 2018; Seddon et al. 2016). The “Third Pole”, comprising the Tibetan Plateau (TP) and Himalaya–Hengduan Mountains (HHMs), constitutes the world's highest alpine ecosystem and contains more ice than anywhere outside the Arctic and Antarctic (Qiu 2008; Liu et al. 2022). As a crucial global biodiversity hotspot, the HHM region harbors the richest alpine flora in the world (Myers et al. 2000). In recent decades, the Third Pole has been experiencing rapid warming, which is causing glacier melting, lake expansion, and the upward shift of the treeline (Gaur et al. 2003; Liu et al. 2021; Tian et al. 2022; Yuke 2019). However, the response of the Third Pole's alpine flora and species to climate change remains largely uncharted.

To adapt to hostile habitats characterized by low temperature, high radiation, and short growing seasons, numerous alpine species inhabit narrow ecological niches and exhibit highly specialized phenotypes (Sun et al. 2014). Alpine species are more sensitive to limiting environmental factors and show less tolerance to conditions change (Engler et al. 2011; Seddon et al. 2016; Yu et al. 2017). In response to climate warming, multiple studies show that plants are shifting upward and northward to track cooler and suitable environments (Ash et al. 2017; He, Burgess, Gao, et al. 2019; Kelly and Goulden 2008; Rana et al. 2022). Conversely, several studies suggest that alpine species are unable to effectively mitigate existential threats via migration (Gottfried et al. 2012; Steinbauer et al. 2018; Nogués‐Bravo et al. 2007). Mountain systems typically exhibit a near‐conical shape, with surface area decreasing as elevation increases, rendering alpine species with “nowhere to go”. With the prevalent upward migration of plant species, local alpine plants are facing competitive replacement by colonizers from lower latitudes (Steinbauer et al. 2018). In a dire scenario, alpine species become trapped at the summits, suffering from compound challenges including habitat degradation, migration constraints, and intensified interspecific competition (Ahmad et al. 2021). Recently, an increasing amount of research has focused on the range shifts of plants within the Third Pole. Several species are projected to maintain or even expand their potential distribution areas, contradicting the “nowhere to go” hypothesis (He, Burgess, Gao, et al. 2019; Liang et al. 2018; You et al. 2018). These studies suggest a possibility that, in the future, the TP may accommodate the migration of species from HHM, but this requires validation through more research on Third Pole species.

Over 8000 species of Lamiaceae plants are widely distributed globally and often endemic, with a significant proportion of monotypic and oligotypic genera among the 226 genera (WCVP; The World Checklist of Vascular Plants). *Elsholtzia eriostachya*, *Eriophyton wallichii*, *Marmoritis complanata*, and *Phlomoides rotata* are four Lamiaceae herbs endemic to the Third Pole. They are concentrated in the HHM's alpine regions above 4000 m a.s.l. and have historically been collected as traditional medicinal herbs. In recent decades, medicinal species in the Third Pole have been under threat from climate change and excessive harvesting (Law and Salick 2005; Niu et al. 2021). While their medicinal properties have been widely studied, few studies have addressed how their distribution will change in response to future climate warming, which is the focus of this study.

In order to evaluate the importance of environmental variables in shaping species distributions and to predict changes in species distributions in response to future climate changes, Ecological Niche Modeling (ENM) has been widely used and has become a standard tool for ecologists and conservation biologists (Peterson and Vieglais 2001). ENM can simulate the potential distribution areas of species based on their actual geographical distribution data and related environmental variables. Currently, the primary algorithms of ENM include generalized linear models (GLM), gradient boosting machines (GBM), classification tree analysis (CTA), artificial neural networks (ANN), one Rectilinear Envelope Similar to BIOCLIM (SRE), flexible discriminant analysis (FDA), random forests (RF), and maximum entropy models (MaxEnt). However, when predicting the potential distribution areas of species, a single algorithm model often lacks stability and exhibits relatively large biases. In contrast, ensemble models such as *biomod2* (v4.2‐5‐2, https://cran.r‐project.org/package=biomod2) R package built on multiple algorithms, tend to perform better (Thuiller et al. 2024). Therefore, the species distribution models used in current species distribution studies are gradually shifting from single models to ensemble models (Araujo and New 2007).

Here, we collected species occurrence records, including specimens, sites in previous studies, and unpublished field observations, to assess the current distribution of four Lamiaceae species. To examine species' responses to climate change, we combined environmental data and applied an ensemble ENM framework to project the potential distribution shifts of species across the past, present, and future (2090; SSP 126 and SSP 585).

Through assessing the future distribution of species, we seek to answer the following questions: (a) What factors primarily shape the distribution patterns of examined species? (b) Whether the potential distribution areas of these plants will decrease or increase in the future? (c) In the future, will the TP be capable of accommodating species migrations from the HHMs? (d) Whether there exist stable suitable habitats that can sustain the long‐term distribution of species under climate change? In addition, while we are more concerned with future trends, we have also reconstructed the historical distributions of species during the Last Interglacial (LIG) and the Last Glacial Maximum (LGM) to better understand the formation of their current range (Clark et al. 2009; Shackleton et al. 2020).

## 2 Materials and Methods

### 2.1 Study Area and Species Occurrence Data

**FIGURE 1:**
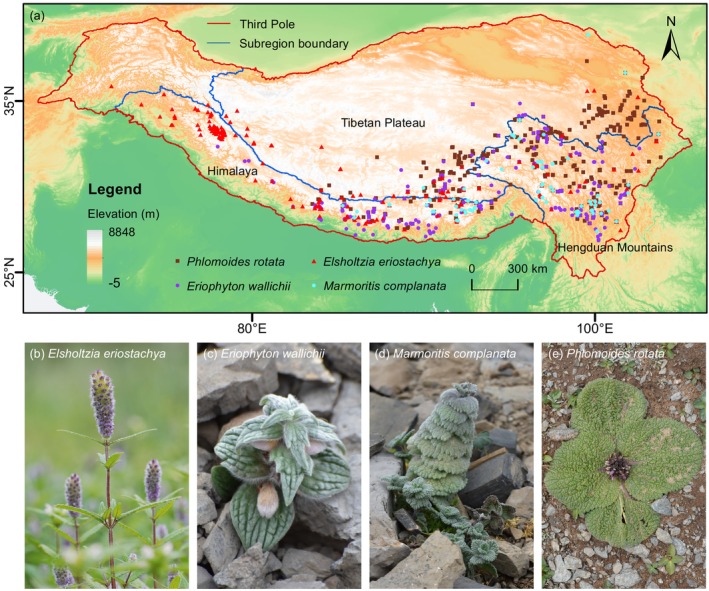
Study area and the occurrence sites of four Lamiaceae species in the Third Pole. Red lines represent the boundary of the Third Pole, and blue lines indicate inner boundary of Tibetan Plateau, Himalaya–Hengduan Mountains.

The study area is within the Third Pole, with its boundaries referenced from the previous study (Liu et al. 2022). The occurrence data for all species were acquired from the Global Biodiversity Information Facility (GBIF), National Plant Specimen Resource Center (NPSRC), Plant Photo Bank of China (PPBC), previous studies (Citations) and field surveys (Table S1, Figure 1). We adopted occurrence records with detailed latitude and longitude information and conducted manual verification based on the recorded locations to ensure authenticity and accuracy. For a few records that have detailed locations but are missing geographical coordinates, we manually obtained these exact coordinates using Google Maps. Raw comprehensive datasets may contain duplicated records and spatial clustering. To reduce spatial sampling bias in the occurrence data, we used the *spThin* R package to perform spatial thinning (Aiello‐Lammens et al. 2015), ensuring that occurrences were at least 5 km apart. Besides, we used the *alphahull* (v.2.5, https://cran.r‐project.org/package=alphahull) R package (Pateiro‐López and Rodríguez‐Casal 2010) to generate the study area with a buffer range of 200 km (VanDer Wal et al. 2009) for each species to avoid overfitting in the following analysis. As a result, 740 occurrence records were used in ENM analysis for four species (Table S2).

### 2.2 Environmental Variables

We gathered climatic, topographic, and edaphic data from various sources, encompassing 26 variables (Table S3). The 19 bioclimatic variables and elevation data were acquired from Worldclim 2.1, which offers both current and future bioclimatic variables, with a spatial resolution of 2.5 arc min, covering the periods of 1970–2000 and 2081–2100 (Fick and Hijmans 2017). We extracted slope and aspect data from the Digital Elevation Model (DEM) using spatial analysis tools in ArcGIS v10.8 (Esri, Redlands, CA, USA). Organic carbon stocks and total nitrogen were sourced from the World Soil Information (WoSIS) database (Batjes et al. 2020). Forest variables were downloaded from the Global Climate Analysis and Modeling (GCAM–Demeter) database (Chen et al. 2020). Additionally, the anthropogenic variable (human footprint index) was obtained from the Human‐Footprint database (Venter et al. 2016). All the environmental variables were resampled to a resolution of 2.5 arc min. To avoid multicollinearity among the variables, which could compromise the reliability of the model, we employed the variance inflation factor (VIF) as an indicator. Using the “vifstep” function from the usdm R package (Naimi et al. 2014), we removed correlated climate factors with a stringent threshold of VIF > 5 (Mambo et al. 2024; Liu et al. 2025), and all variables are listed in Table S3. Then, the environmental variables were extracted based on the study area generated by the *alphahull* R package (Pateiro‐López and Rodríguez‐Casal 2010). We calculated the primary climatic characteristic values for different subregions and periods to represent the trends of climate change, which are presented in Table S4.

### 2.3 Ecological Niche Modeling

The thinned occurrence data (Table S2) and retained variables (Table S3) for each species were used to conduct ENM analysis using *Biomod2* (Thuiller et al. 2009) based on 10 models (Table S5) from the LIG (116–129 ka) (Shackleton et al. 2020), LGM (18–26.5 ka) (Clark et al. 2009), current, and future (2090). For future scenarios, we used two shared socioeconomic pathways (SSPs): SSP 126 (with 2.6 W/m^2^ by the year 2100) and SSP 585 (with an additional radiative forcing of 8.5 W/m^2^ by the year 2100), to explore the potential distribution areas in the future (2090). The modeling steps were as follows: The bootstrap resampling method was used; 75% occurrence data for calibrating the models and the rest of the occurrence data (25%) for validating the models; the running using 10 replicates; model performance was considered reliable based on two metrics: the threshold‐independent area under the curve (Taberlet et al. 1998) values ≥ 0.9 and the threshold‐dependent true skills statistic (TSS) values ≥ 0.7 (Table S6).

### 2.4 Potential Distribution Area Estimation

We visualized the model‐generated data using ArcGIS v10.8 (Esri, Redlands, CA, USA). Based on the suitability thresholds derived from the model, we categorized the suitability of the habitat of these species into three levels: nonsuitable area (0), suitable area (0–1), and highly suitable area (0.75–1), using the “Reclassify” function in ArcGIS v10.8 (Esri, Redlands, CA, USA). Subsequently, we calculated and mapped the spatial distribution shifts of these species from the current to the future (2090) under the scenarios SSP 126 and SSP 585 based on the suitable areas (0–1). To further explore the stable potential distribution areas crossing different periods (LGM–Current–2090), we overlapped the highly suitable areas (0.75–1) of ENM results from LGM, current, and future (2090), using the “Extract by Mask” function in ArcGIS v10.8 (Esri, Redlands, CA, USA).

## 3 Results

### 3.1 Modeling Evaluation and Climate Factor Importance

All the ensemble models for species considered show high consistency and reliability, with AUC (area under the curve) values ≥ 0.9 and TSS (True Skill Statistics) values ≥ 0.5, which are better than a random model (AUC = 0.5) (Table S6).

**FIGURE 2:**
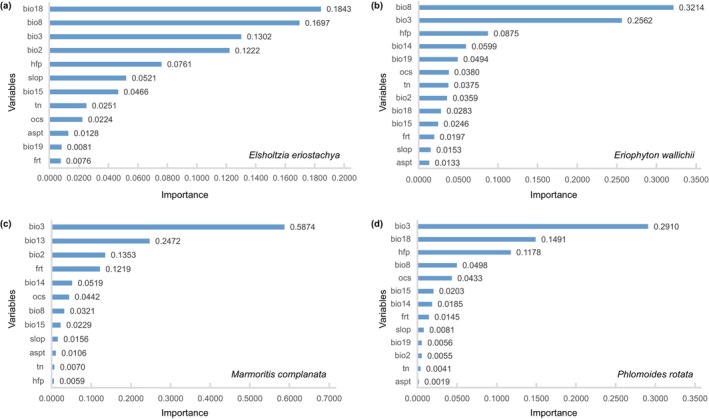
Estimates of the relative contributions of the environmental variables for the four Lamiaceae species in the Third Pole.

The ENMs indicate that all species are significantly influenced by the temperature‐related variables, precipitation‐related variables, and considerably influenced by hfp to varying degrees (Figure 2). Isothermality (BIO3) was the decisive factor for predicting the distribution of *Marmoritis complanata* (0.58) and *Phlomoids rotata* (0.29); Whereas Mean Temperature of Wettest Quarter (BIO8) was the most important variable for *Eriophyton wallichii*. Precipitation of Warmest Quarter (BIO18) was the most important variable for *Elsholtzia eriostachya*.

### 3.2 Current Potential Habitat Areas and Range Dynamics

**FIGURE 3:**
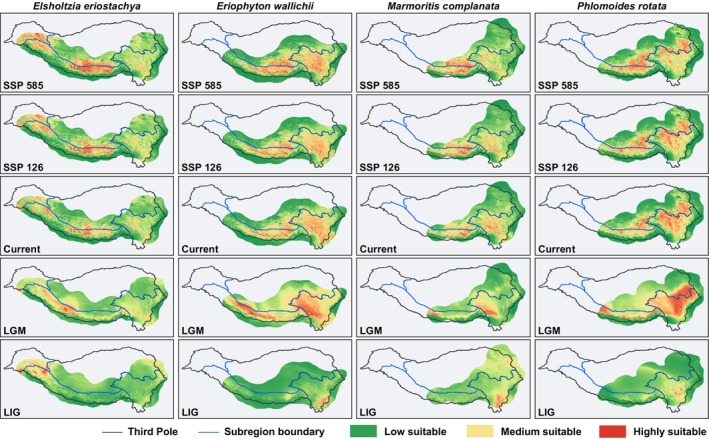
Potential distribution ranges for four Lamiaceae species from the past to the future in the Third Pole. The color gradient from green to red represents low to high suitability. LIG, Last Interglacial (116–129 ka); LGM, last glacial maximum (18–26.5 ka); SSP 126 and SSP 585 represent two shared socioeconomic pathways (SSPs) in the year 2090.

The current potential distribution areas estimated by ENM indicate that all the species are primarily distributed in the Third Pole with mean elevations exceeding 4000 m a.s.l. (Table 1). The spatial size of the potential distribution areas for *Elsholtzia eriostachya*, *Eriophyton wallichii*, and *Phlomoides rotata* is similar, each exceeding 48 km^2^. In contrast, the potential distribution area of *Marmoritis complanata* is relatively smaller, encompassing 20 km^2^ (Table 2). Mountain systems are still the primary distribution areas, and they also form substantial populations in the regions adjacent to the Himalaya –Hengduan Mountains on the TP. *Eriophyton wallichii*, *Phlomoides rotata*, and *Marmoritis complanata* are distributed in both the Himalayas and the Hengduan Mountains. *Elsholtzia eriostachya*, concentrated in the Himalayas, has only scattered distributions in the Hengduan Mountains (Figure 3).

**TABLE 1:** Average elevation (m) of ensemble model projections in the different periods.

Species	*Elsholtzia eriostachya*	*Eriophyton wallichii*	*Marmoritis complanata*	*Phlomoides rotata*
LGM	3916	4217	4533	4238
Current	4199	4297	4564	4311
2090 (SSP 126)	4342	4385	4673	4367
2090 (SSP 585)	4516	4566	4773	4441
Stable area in LGM–current	3849	4218	4554	4207
Stable area in current–SSP126	4281	4357	4623	4324
Stable area in current–SSP585	4409	4515	4693	4350

**TABLE 2:** Range size of ensemble model projections in the different periods.

Species	Period	Lost area (km^2^)	Gained area (km^2^)	Stable area (km^2^)	Percentage of loss	Percentage of gain	Area change
*Elsholtzia eriostachya*	LGM–Current	274,271	311,823	173,837	61.21%	69.59%	Expansion
	Current–SSP 126	39,445	159,184	446,215	8.12%	32.78%	Expansion
	Current–SSP 585	91,094	291,615	394,566	18.76%	60.05%	Expansion
*Eriophyton wallichii*	LGM–Current	484,531	100,572	389,705	55.42%	11.50%	Constriction
	Current–SSP 126	73,906	42,257	416,371	15.07%	8.62%	Constriction
	Current–SSP 585	95,625	95,973	394,652	19.50%	19.58%	Expansion
*Marmoritis complanata*	LGM–Current	118,246	144,253	65,226	64.45%	78.62%	Expansion
	Current–SSP 126	32,396	48,802	177,083	15.47%	23.30%	Expansion
	Current–SSP 585	61,979	102,395	147,500	29.59%	48.88%	Expansion
*Phlomoides rotata*	LGM–Current	326,389	132,292	376,215	46.45%	18.83%	Constriction
	Current–SSP 126	52,466	38,507	456,041	10.32%	7.57%	Constriction
	Current–SSP 585	126,233	67,257	382,274	24.82%	13.23%	Constriction

**FIGURE 4:**
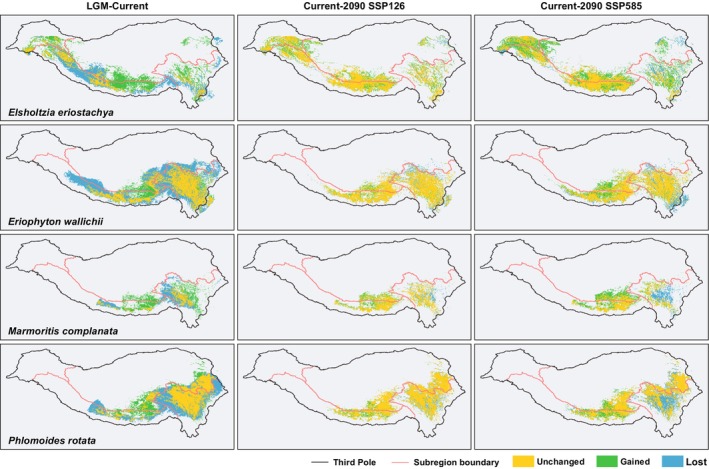
Distribution change patterns of four Lamiaceae species across different periods in the Third Pole. The yellow region shows the area remained still (Unchanged), the green region shows the new area to be colonized (Gained), and the blue region shows the area to be lost (Lost).

Simulations indicate that the species' potential distribution areas shifted upward and northward, expanding significantly from LIG to LGM. During the LGM, the species held continuous potential distribution areas along the HHM corridors. Following the post‐LGM, the Third Pole has undergone overall warming and an increase in precipitation (Table S4); the suitable habitats for the species migrated toward higher elevations (Table 1). Consequently, their low‐elevation distributions disappeared, resulting in isolated distributions in the Himalayas and Hengduan Mountains (Figures 3 and 4). Meanwhile, the species have acquired new distribution areas and successfully established populations on the TP. From LGM to current period, the diffusion of *Elsholtzia eriostachya* and *Marmoritis complanata* has been accompanied by significant distribution area migration, with over 60% loss and new acquisition. The distribution changes of *Eriophyton wallichii* and *Phlomoides rotata* are less pronounced, but they exhibit a contraction trend, with at least 27% lost.

Overall, in the future, the Third Pole is projected to continue experiencing warming and increased precipitation. Under worse scenarios, the trend is likely to be exacerbated (Table S4). Species' potential distribution areas will expand in the TP while contracting in the regions of the Hengduan Mountains (Figures 3 and 4). All species are facing a reduction in their current potential distribution range of at least 8% up to as much as 30%. Species with a higher current mean elevation exhibit more pronounced distribution losses in the Hengduan Mountains (Tables 1 and 2). Warmer scenarios will accelerate the shifts with higher loss and gain.

Based on predictions, all species are projected to shift toward higher elevations, with greater elevational shifts occurring under scenario SSP 585. Under the scenario SSP 126, species will shift upward by ca. 1.5 m per year, resulting in a cumulative migration of 99 m by 2090. While under the scenario SSP 585, species will experience an average upward shift of 230 m, exceeding 3 m per year. The distances of upward shift vary among species. Under the worse climate scenario, the mean elevation of *Elsholtzia eriostachya* increased by 317 m, while *Phlomoides rotata* increased by 130 m (Table 1).

The loss of current habitats, coupled with the acquisition of potential habitats, is leading to different future trends among species. The potential distribution area of *Elsholtzia eriostachya* and *Marmoritis complanata* will increase in the future, while *Phlomoides rotata* will decrease. Noteworthy, the potential distribution area of *Eriophyton wallichii* contracts under scenario SSP 126 while expanding under SSP 585. Under the warmer scenario, *Eriophyton wallichii* will shift more toward higher elevations and latitudes, resulting in newly acquired potential distribution areas on the TP far exceeding those lost. In contrast, the distribution of *Elsholtzia eriostachya* expanded significantly with the most upward shifts (Expanding by 25% under SSP 126, 41% under SSP 585), *Phlomoides rotata* shrunk with the least upward shifts (Shrinking by 3% under SSP 126, 12% under SSP 585). Compared with losing 19% of its current distribution, acquiring over 60% of new distribution has led to the greatest expansion of *Elsholtzia eriostachya* among all species (Table 2).

### 3.3 Potential Stable Distribution Area

**FIGURE 5:**
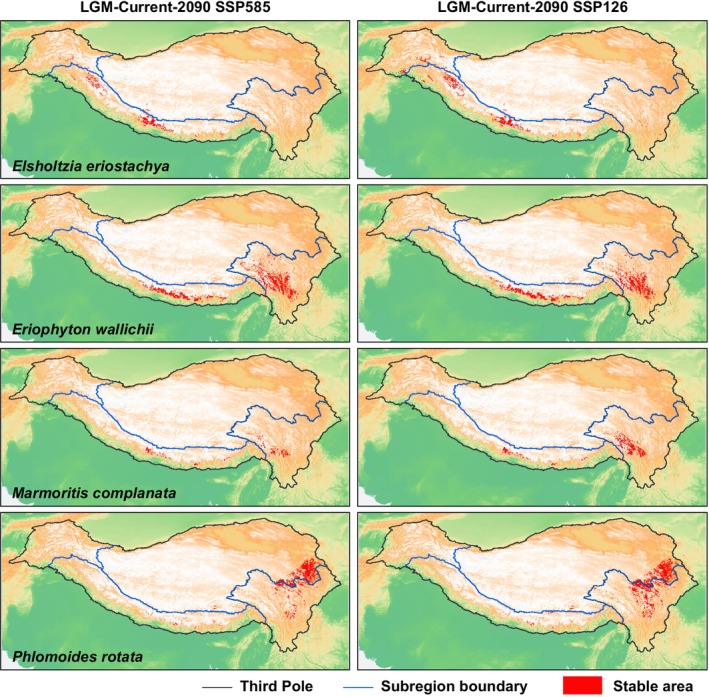
Stable potential distribution areas of four Lamiaceae species across different periods in the Third Pole. The red area refers to the intersection of the highly suitable area (suitability at the range of 0.75 to 1) in different periods.

From the LGM to the present, and under different future climate scenarios, certain regions have consistently provided stable, highly suitable habitats (0.75–1) for plant survival across different periods (Figure 5). Based on ENM, *Eriophyton wallichii* and *Marmoritis complanata* exhibit stable areas both in the Himalayas and Hengduan Mountains regions. For *Elsholtzia eriostachya*, the area is concentrated in the Himalayas. For *Phlomoides rotata*, the area is primarily distributed in the Hengduan Mountains and the adjacent TP, with only sporadic occurrences in the Himalayas. Although it currently has a substantial distribution in the Himalayas and adjacent TP regions, the population in this region was established after the LGM. The high‐suitability habitats in the Himalayas did not persist stably through long‐term climate change. As a result, the stable area of *Phlomoides rotata* is located at elevations lower than the current average elevation of its overall distribution. In contrast, the stable areas of the remaining species are generally situated at elevations higher than their current average elevation. Moreover, under warmer scenarios, the contraction of stable areas toward higher elevations is accelerated, resulting in a widespread reduction in their size (Table S7).

## 4 Discussion

### 4.1 Contribution of Environmental Variables

Consistent with studies on other alpine plants, we found that temperature and precipitation play a decisive role in determining the distribution range of examined species. Alpine plants have evolved a variety of distinctive traits to adapt to hostile habitats; many of these specialized traits have been proven to enhance plants' adaptation to low‐temperature conditions, such as trichomes, glasshouse bracts, and so forth (Peng et al. 2015; Song et al. 2013, 2015; Sun et al. 2014). Due to their high specialization to extremely low temperatures, alpine plants are more sensitive to warming. Temperature has become a key limiting factor for many alpine species, shaping the distribution range and driving the range shifts (Dolezal et al. 2016). Most examined species exhibit typical cold adaptation traits, like dense trichomes and low height. The assessment of factors importance reveals that species are strongly influenced by temperature‐related factors, especially Bio3 (Isothermality). Isothermality indicates the stability of regional temperature, derived from the annual mean diurnal range (Bio2) and annual temperature range (Bio7), and quantifies the oscillations of day‐to‐night temperature relative to the summer‐to‐winter temperature variations. Previous studies have shown that isothermality significantly influences plant distribution at the global scale, affecting both the size of the distribution range and the latitudinal boundaries (Huang et al. 2021). In alpine regions, lower isothermality has been shown to lead to pollinator limitation, which in turn restricts population dispersion (Miladin et al. 2022). The distribution of many alpine plants is significantly influenced by isothermality (Rana et al. 2022; You et al. 2018; Yu et al. 2017), indicating that alpine plants are more susceptible to temperature limitations. In our study, the overall increase in isothermality in the Third Pole (Table S4), accompanied by the northward expansion of species distribution, is consistent with the results of previous studies (Huang et al. 2021).

Besides temperature, precipitation‐related factors are also common dominant factors influencing alpine plant distribution. Especially for subnival species, water resources mainly consist of snowmelt and precipitation (Jonas et al. 2008). During the warm growing season, it is rare to experience temperature limitations, and precipitation may become the primary limiting factor (Wang et al. 2020). The examined species, except *Elsholtzia eriostachya*, are distributed in the subnival belt, the scree below the snow cover, characterized by colder temperatures that render them more vulnerable to temperature constraints, and precipitation is not the dominant factor. For *Elsholtzia eriostachya*, which is primarily distributed in lower‐elevation grasslands, temperature limitations are less of a concern. Instead, precipitation of the warmest quarter (Bio8) exerts a greater influence on this species.

It is noteworthy that the impact of human activities on alpine plants deserves greater attention. Recent studies have demonstrated that human activities have the capacity to alter the phenotypic traits of species, as well as affect changes in plant distribution areas (Xu et al. 2019). Commercial harvesting has driven the evolution of camouflage adaptations in *Fritillaria* sp. and a reduction in height of *Saussure* sp., both of which are medicinal alpine plants (Law and Salick 2005; Niu et al. 2021).

### 4.2 Historical Shifts and Current Pattern

The historical distribution reconstructed based on ENM indicates that the examined species experienced a significant expansion from LIG to LGM, with a continuous distribution spanning the HHM region. Following the LGM, as temperature increased (Table S4), the species' range contracted toward higher elevations and latitudes, resulting in fragmentation and the emergence of geographical barriers.

Over the past millions of years, there has been continuous biological interchange between the Hengduan Mountains and the Himalayas. Climate oscillations have led to massive species extinctions but have also increased the frequency of species migration, especially from the Hengduan Mountains to the Himalayas, which has rendered the flora of the two regions highly similar (Xing and Ree 2017; Yu et al. 2020). Alpine plants have undergone repeated migrations, as well as habitat contraction and expansion, in response to glacial–interglacial cycles. During the LGM, the Third Pole experienced significantly colder temperatures and ice sheet expansion, leading to species local extinction or survival in refugia (Liu et al. 2014; Mao et al. 2021; Schneider Von Deimling et al. 2006). In contrast, this period provided favorable conditions for the expansion of some alpine species, facilitating their migration into newly available habitats at lower elevations (Luo et al. 2017; Rana et al. 2022; You et al. 2018). Taking the experimental species as an example, a continuous high‐suitability area was formed along the HHM corridor, facilitating the exchange and diffusion of species among the HHM.

As the glacial periods ended and temperatures rose, alpine plants contracted back to higher latitudes and elevations (Birks 2008; Liu et al. 2023; Petit et al. 2008; Pu et al. 2024). With the loss of low‐elevation areas, the habitats of species become increasingly fragmented, and geographical barriers intensify population differentiation. The Ward Line–Mekong–Salween Divide, a classic geographical barrier, has been proven to drive the phylogeographical differentiation of *Marmoritis planatum* (Luo et al. 2017). Our study reveals that the current potential distribution area of species in the Hengduan Mountains tends to be isolated, lacking direct connectivity with other potential distribution areas. Mountain ridges and river valleys serve as geographical barriers, segmenting the current potential distribution areas of species (Li et al. 2021). Our results suggest that climate warming will accelerate the emergence of new geographical barriers within the existing potential distribution areas of alpine species.

### 4.3 Future Pattern

Consistent with predictions for other alpine plants, ENM forecasts that all the examined species will shift toward higher latitudes and elevations in the future. A warmer climate will accelerate and intensify the movement of alpine species to track cooler, more suitable habitats. Additionally, the TP is projected to transform substantial newly available potential distribution areas under future warm scenarios.

Based on species records and model predictions, previous publications show that alpine species have been shifting to higher elevations over the past few decades, and they are expected to continue shifting upward to track climate warming (He, Burgess, Yang, et al. 2019; Telwala et al. 2013). However, upward shifts may result in habitat contraction. Typically, the curve of land surface area versus elevation in mountain systems exhibits a unimodal pattern. It is generally accepted that land surface area decreases with increasing elevation toward summits, which means suitable habitats for alpine species will shrink as they migrate upward (Dullinger, Gattringer, et al. 2012; Engler et al. 2011; Loarie et al. 2009). However, an unconventional expansion of suitable areas often occurs as plants shift upward in the Third Pole (He, Burgess, Gao, et al. 2019; He, Burgess, Yang, et al. 2019; Rana et al. 2022; You et al. 2018). The TP boasts extensive potential available land at higher elevations, serving as an escape region for alpine species from the adjacent HHM mountains (Liang et al. 2018; Liu et al. 2022). Under future climate warming, warmer temperature and increased precipitation will transform currently unsuitable regions, such as permafrost, into suitable habitats for alpine species. Ideally, during migration, species' ranges will expand due to the newly gained suitable distribution areas on the TP exceeding those lost in the HHM. For instance, despite having suffered the highest loss of the current areas in HDM, *Marmoritis complanata* has overcompensated by gaining new areas on the TP. However, species that lack sufficient new suitable habitats on the TP in the future will be confined to mountainous regions and experience range contraction. In the case of *Marmoritis complanata*, only under warmer scenarios can it gain more suitable areas on the TP to offset the losses in the HHM. *Phlomoides rotata* will inevitably experience contraction in any scenario, with minimal gains and higher losses of areas in mountainous regions. The availability of larger new potential suitable areas serves as a safeguard for species against climate risks. We suggest that the TP will be a key destination for species migration from adjacent mountains, with potential for extensive areas to be converted into suitable habitats. However, when considering the HHM alone, the situation aligns with the “nowhere to go” hypothesis, suggesting that suitable habitats will shrink as alpine species move upward.

### 4.4 Risks Underlying Shift

Although predictions suggest that the potential distribution areas of individual species may expand, the future of these species remains uncertain due to the accumulation of risks from multiple aspects. There may be overly optimistic estimations regarding the future situation of alpine species. The optimistic predictions imply that species need to fully track climate change. It is almost impossible to occupy all newly predicted suitable areas, while most species will experience various levels of contraction in their current suitable areas, which poses an urgent threat to the species. Migration lag frequently occurs in alpine species due to their limited dispersive ability, ultimately resulting in incomplete filling of the predicted range (Dullinger, Willner, et al. 2012; Morgan and Venn 2017). Features such as shorter height, less efficient vectors, and lower reproductive investment result in their limited dispersal abilities. As a reference, most alpine species in the Snowy Mountains have a dispersal distance of less than 10 m (Morgan and Venn 2017). Geographical barriers, habitat fragmentation, and limited dispersal abilities have led to a persistent lack of gene flow among alpine species populations, resulting in considerable genetic divergence (Luo et al. 2017).

The ability of species to track climate change varies significantly, with alpine species often lagging while others successfully track their ecological niches (Tomiolo and Ward 2018). In the European Alps, non‐native species are spreading upward more rapidly than native alpine species. Species originating from lower elevations may possess greater competitive advantages, benefiting more from climate warming due to their faster growth rates and stronger dissemination capabilities (Dainese et al. 2017). The overall upward shift of species is driving an increase in species richness at summits, reshaping community composition and biotic interactions. As interspecific competition intensifies, cold‐tolerant, slow‐growing native alpine species may be competitively replaced by migrant species (Steinbauer et al. 2018). Beyond direct interspecific competition, the increased contact between lowland and alpine species enhances the possibility of interspecific hybridization. Furthermore, genetic swamping may ultimately result in the genetic extinction of alpine species (Gómez et al. 2015).

Based on the preceding context, the outcomes of alpine plants tracking climate change may not be as optimistic as anticipated. Therefore, conserving stable, suitable habitats becomes even more critical. These high‐elevation stable areas in the HHMs (Figure 5) will serve as refugia, providing stable habitats for species to survive during climate warming.

## 5 Conclusion

Climate shapes plant distributions, and alpine plants on the Third Pole have undergone repeated upward and downward migrations along mountain systems in response to climatic fluctuations (Birks 2008). While alpine plants have adapted to harsh habitat conditions, temperature and precipitation‐related climatic factors have become limiting factors for their survival (Dullinger, Willner, et al. 2012; Sun et al. 2014). From occupying continuous distribution areas at lower elevations of the HHMs during the LGM to the ongoing upward contraction of their ranges since the post‐LGM period and into the future, their distributions in the HHM are experiencing inevitable contraction during upward migration, yet are expected to gain substantial new potential distribution areas transformed by climate change on the TP.

Our study provides novel insights into the differential climate‐mediated biogeographic responses across distinct ecological subregions of the Third Pole. The ecosystems of the Third Pole are undergoing profound climate‐mediated reorganization. Recent decades have witnessed a widespread vegetation greening trend across the TP (Xu et al. 2023), where climatic changes are restructuring local communities and facilitating the upward migration and range expansion of species from adjacent mountain systems (Liang et al. 2018). Nevertheless, the concurrent warming in mountain ecosystems has triggered substantial habitat loss and localized extinctions for numerous species. Broad‐scale studies of species' climatic responses may overlook the heterogeneity of ecological processes across different ecosystem subregions and obscure the critical extinction debts that are progressively accumulating in these vulnerable mountain ecosystems. In planning biodiversity conservation and management practices, high‐elevation stable areas in HHM should be given greater emphasis.

## Author Contributions

**Shou‐Kui Wang:** conceptualization (lead). **Zhi‐Peng Li:** formal analysis (lead), methodology (lead), visualization (lead), writing – review and editing (equal). **Rui Wu:** data curation (lead), investigation (lead), writing – original draft (equal). **Hai‐Ling Qi:** methodology (supporting), validation (supporting), visualization (supporting). **Hong Ke:** data curation (equal), investigation (equal). **Xiong‐Hui Huang:** data curation (equal), investigation (equal). **Ji‐Hua Zhou:** data curation (equal), investigation (equal). **Yong Tang:** data curation (equal), investigation (equal). **Jiang‐Hua Ran:** data curation (equal), investigation (equal). **Yong‐Qian Gao:** conceptualization (lead), supervision (lead).

## Conflicts of Interest

The authors declare no conflicts of interest.

## Supporting information


**Table S1.** The occurrence data resource database and websites.
**Table S2.** Occurrence sites used for ENM analysis.
**Table S3.** The environmental variables.
**Table S4.** Mean values of three environmental variables in three subregion of Third Pole in different periods.
**Table S5.** Ten models are used to conduct Ecological niche modeling.
**Table S6.** Ensemble model performance for four species.
**Table S7.** The average elevation (m) and range size (km^2^) of the stable potential distribution areas across different periods.

## Data Availability

The data that supports the findings of this study are available in the Supporting Information of this article.
